# Establishment of patient-derived xenograft models and cell lines for malignancies of the upper gastrointestinal tract

**DOI:** 10.1186/s12967-015-0469-1

**Published:** 2015-04-11

**Authors:** Helene Damhofer, Eva A Ebbing, Anne Steins, Lieke Welling, Johanna A Tol, Kausilia K Krishnadath, Tom van Leusden, Marc J van de Vijver, Marc G Besselink, Olivier R Busch, Mark I van Berge Henegouwen, Otto van Delden, Sybren L Meijer, Frederike Dijk, Jan Paul Medema, Hanneke W van Laarhoven, Maarten F Bijlsma

**Affiliations:** Laboratory for Experimental Oncology and Radiobiology, Academic Medical Center, Meibergdreef 9, Amsterdam, AZ 1105 The Netherlands; Department of Surgery, Leiden University Medical Center, Albinusdreef 2, Leiden, ZA 2333 The Netherlands; Department of Surgery, Academic Medical Center, Meibergdreef 9, Amsterdam, AZ 1105 The Netherlands; Department of Gastroenterology and Hepatology, Academic Medical Center, Meibergdreef 9, Amsterdam, AZ 1105 The Netherlands; Department of Pathology, Academic Medical Center, Meibergdreef 9, Amsterdam, AZ 1105 The Netherlands; Department of Radiology, Academic Medical Center, Meibergdreef 9, Amsterdam, AZ 1105 The Netherlands; Department of Medical Oncology, Academic Medical Center, Meibergdreef 9, Amsterdam, AZ 1105 The Netherlands

**Keywords:** Biobank, Patient derived xenograft, Esophageal cancer, Pancreatic cancer, Paracrine signaling, Hedgehog

## Abstract

**Background:**

The upper gastrointestinal tract is home to some of most notorious cancers like esophagogastric and pancreatic cancer. Several factors contribute to the lethality of these tumors, but one that stands out for both tumor types is the strong inter- as well as intratumor heterogeneity. Unfortunately, genetic tumor models do not match this heterogeneity, and for esophageal cancer no adequate genetic models exist. To allow for an improved understanding of these diseases, tissue banks with sufficient amount of samples to cover the extent of diversity of human cancers are required. Additionally, xenograft models that faithfully mimic and span the breadth of human disease are essential to perform meaningful functional experiments.

**Methods:**

We describe here the establishment of a tissue biobank, patient derived xenografts (PDXs) and cell line models of esophagogastric and pancreatic cancer patients. Biopsy material was grafted into immunocompromised mice and PDXs were used to establish primary cell cultures to perform functional studies. Expression of Hedgehog ligands in patient tumor and matching PDX was assessed by immunohistochemical staining, and quantitative real-time PCR as well as flow cytometry was used for cultured cells. Cocultures with Hedgehog reporter cells were performed to study paracrine signaling potency. Furthermore, SHH expression was modulated in primary cultures using lentiviral mediated knockdown.

**Results:**

We have established a panel of 29 PDXs from esophagogastric and pancreatic cancers, and demonstrate that these PDXs mirror several of the (immuno)histological and biochemical characteristics of the original tumors. Derived cell lines can be genetically manipulated and used to further study tumor biology and signaling capacity. In addition, we demonstrate an active (paracrine) Hedgehog signaling mode by both tumor types, the magnitude of which has not been compared directly in previous studies.

**Conclusions:**

Our established PDXs and their matching primary cell lines retain important characteristics seen in the original tumors, and this should enable future studies to address the responses of these tumors to different treatment modalities, but also help in gaining mechanistic insight in how some tumors respond to certain regimens and others do not.

**Electronic supplementary material:**

The online version of this article (doi:10.1186/s12967-015-0469-1) contains supplementary material, which is available to authorized users.

## Background

Despite the progress in both scientific understanding and treatment of most tumor types, some malignancies have remained notoriously difficult to treat. Good examples of such difficult cancers are those of the upper gastrointestinal (GI) tract, including esophagogastric and pancreatic adenocarcinoma. The incidences of these tumors are increasing in the Western world and given that their survival rates are not significantly improving, they are within the leading causes of death by 2030 [1]. A need to better understand these malignancies and to develop models that address the crucial features that render them so lethal is therefore apparent.

Contributing to the lethality of these cancers is the large degree of heterogeneity observed. The clinical implications of this heterogeneity are multiple; first, sampling or imaging part of the tumor to assess the biology driving its growth is not necessarily representative of the tumor bulk (intratumor heterogeneity) or tumors in other patients (intertumor heterogeneity). Treatment decisions based on this can thus be inadequate. Second, different populations within or between tumors can differ in sensitivity to the drug already at the start of treatment, or display different rates at which resistance develops.

As a consequence, tissue banks that hold large sample numbers to cover the breadth of this diversity are required to measure relevant parameters, and models to manipulate and perform functional experiments should be generated to reflect the heterogeneity observed in the patients. These considerations explain why genetically engineered mouse models (GEMMs) are inadequate to mimick broad, unselected patient populations; they are driven by a very well defined set of driver mutations which can never reflect the diverse spectrum of genetic aberrations found in human tumors. Another important concern is that the time within which these tumors arise in mice does not allow for the ‘evolution’ observed in patient derived tumors. In addition, for some diseases like esophagogastric cancer, appropriate GEMMs are not available making the rationale to invest in a viable tissue biobank, including patient derived xenograft (PDX) models and primary cell lines to study tumor biology, even more apparent.

One of the key pathways for normal gastrointestinal development, the Hedgehog signaling pathway, is also involved in the formation and progression of malignancies of the upper GI tract [2]. As a consequence, it is being considered as a therapeutic target in a broad range of tumors [3,4]. The pathway is activated by binding of Hedgehog ligands (SHH, IHH, or DHH) to their receptor Patched (PTCH), which then alleviates the inhibitory function of PTCH on the activating receptor Smoothened (SMO). This in turn leads to activation of downstream mediators, such as the Gli transcription factors, that orchestrate the transcriptional response and functional outcome of pathway activation.

As cells of the pancreas move through the different stages of malignant progression towards PDAC, they express increasing amounts of SHH [5]. This ligand is notably absent from both the developing as well as the healthy adult pancreas, but its aberrant expression in the pancreas has been shown to drive tumor progression, and inhibition of its downstream signaling pathway is effective in some preclinical models for PDAC [6,7]. For EAC similar data have been reported [8,9], but knowledge on the role of Hedgehog proteins in cancers of this organ is much more limited compared to PDAC. Interestingly, Hedgehog ligands have been described to be expressed in healthy esophagus and are required for the homeostasis of this organ [10]. This implies that the expression of Hedgehog ligands in cancer tissue is not exclusive to epithelial cells that have never expressed these ligands, but also that the response to these ligands in a tumor setting is not exclusive to cells that are Hedgehog-naive.

We describe in this paper the establishment of a panel of 29 relevant PDXs from esophagogastric and pancreatic cancers, and show that these PDXs reflect the histological and biochemical characteristics of the original cancer. Matching primary cell lines from the PDX models can be genetically manipulated and used to further study tumor biology and signaling capacity. These models should prove valuable for profiling approaches and detailed functional studies requiring living tissue. In addition, we demonstrate an active (paracrine) Hedgehog signaling mode by both tumor types, the magnitude of which has not been compared directly in previous studies.

## Methods

### Ethics statements

Both the collection and storage of patient material were approved by the institute’s medical ethical committees, and performed according to the guidelines of the Helsinki Convention. Informed written consent was obtained from all patients. Animal work was performed according to protocols approved by the animal experiment ethical committee (DTB102348, LEX102774). All surgical procedures were performed under isoflurane anesthesia.

### Pathology

The surgical resection specimen was inspected and processed according to national and international guidelines [11]. The microscopic assessment was performed by an experienced GI-pathologist and the final diagnosis was set in accordance with the WHO classification [12]. Adenocarcinomas and squamous cell carcinomas were classified according to site of origin and tumor stage, in accordance with the pTNM classification of malignant tumors [13].

### Immunohistochemistry

Patient tumor material or xenografts were fixed in 4% formalin prior to paraffin embedding. Sections of 5 μm were prepared on a microtome. Tissue sections were deparaffinized and heat mediated antigen retrieval was performed using Tris-EDTA buffer solution pH 9 for Hedgehog staining or 10 mM sodium citrate solution pH 6 for other stainings. Endogenous peroxidase activity was blocked with 3% hydrogen peroxide in PBS. Aspecific staining was blocked using Ultra-V Block (Immunologic) for 10 min at room temperature. Primary antibodies were diluted in normal antibody diluent (KliniPath), applied on tissue sections and incubated overnight at 4°C in a humidified chamber. For amplification of signal Brightvision + post antibody block (Immunologic) was used prior to the addition of the secondary antibody; poly-HRP-anti Ms/Rt/Rb IgG (Immunologic) both for 30 min at room temperature. Visualization was performed using Vector® NovaRED™ (Vector Labs) according to manufacturer’s protocol, counterstained with 30% haematoxylin and tissue sections were mounted with non-aqueous medium. Antibodies used for immunohistochemistry were: anti-alpha smooth muscle actin (Abcam, 1:1000); anti-Hedgehog (clone H160, Santa Cruz, 1:1500), anti-Cytokeratin 19 (clone RCK108, BioGenex, 1:1000), anti-pan cytokeratin (clone AE1/AE3, Dako, 1:100).

### Tumor and cell line xenografting

Freshly excised tumor pieces (approx. 3×3×3 mm) originating from primary tumor or liver metastasis were washed several times in PBS containing 10 μg/ml gentamycin (Lonza) and 1% Penicillin-Streptamycin. Pieces were grafted subcutaneously into the flank of immunocompromised NOD.Cg-*Prkdc*^*scid*^*Il2rg*^*tm1Wjl*^/SzJ (NSG) mice (JAX 005557) with Matrigel (BD). Animals were bred and maintained at the local animal facility according to the legislation and ethical approval was obtained for the establishment of patient derived xenografts (PDX). Monitoring for tumor take was done up to 9 months after transplantation. If no tumor was palpable on animals after this period, grafting was considered to be not successful. After outgrowth of patient tumor and reaching a size of approximately 500 mm^3^, PDX tumors were harvested and passaged, and/or used to establish *in vitro* cultures. Tumors were typically retransplanted three times (i.e. up to p4). To establish xenograft tumors from isolated EAC007B cell line, 10^6^ cells were injected into the flank of NSG mice in PBS with 50% Matrigel. For orthotopic injection of PC053M cells into the pancreas, mice received pre-operative analgesics by subcutaneous administration of meloxicam (1 mg/kg) and were operated under isoflurane anesthesia (0.5-2.5% in 100% oxygen). Briefly, a small incision was made in the abdominal skin and peritoneal wall. Thereafter, pancreas was gently pulled out and 10^6^ PC053M cells in 50 μl PBS + 5% Matrigel were injected using a 1 ml syringe and 25G needle. After placing back the pancreas into the abdominal cavity, both muscle and skin layers were closed by surgical suture.

### Isolation and propagation of primary cultures

Harvested xenografts were minced, placed in 8% FBS containing IMDM with collagenase IV (0.5 mg/ml, Sigma) in a tube and incubated at 37°C for 60 min with vortexing every 10 min. The dissociated suspension was passed through a 70 μm strainer to obtain single cells and washed with culture medium. Cell aggregates retained on top of the filter were put in a separate dish. Isolated cells and aggregates were grown in IMDM containing 8% FBS. Purity of the epithelial culture was assessed by flow cytometry with FITC labelled human specific EpCAM antibody staining (DAKO, F0860 at 1:100). For selective trypsinization, cultures were washed twice with PBS, followed by 2–3 min incubation with 0.05% Trypsin/0.02% EDTA solution at 37°C. Detached cells were gently washed away with 8% serum containing medium and selective removal of fibroblast was repeated once cells reached confluence.

### Flow cytometry

Cell were harvested with trypsin-EDTA solution (Lonza) and washed in FACS buffer (PBS with 1% FBS). Staining was performed with hybridoma supernatant containing either anti-SHH antibody clone 5E1 (0.08 μg/ml) or anti-Myc antibody clone 9E10 (1 μg/ml), diluted 1:5 in FACS buffer for 30 min at 4°C. Secondary APC labeled anti-mouse antibody (BD, 550826) was used at a 1:500 dilution. After washing, cells were resuspended in FACS buffer containing 1 μg/ml propidium Iodide (PI, Sigma) and acquired on a FACSCanto II (BD, Franklin Lakes, NJ). In case of the EAC007B line, cells were costained with FITC labelled anti-human EpCAM antibody to allow for exclusion of mouse fibroblasts from the analysis (DAKO, F0860 at 1:100). Data were analyzed with FlowJo 7 (Tree Star, Ashland, OR).

### Hedgehog reporter assay

Mouse embryonic fibroblasts stably transduced with the GBS-GFP reporter construct (GGM cells, [21]) were grown under standard cell culture conditions in high glucose DMEM (Lonza) containing 8% FBS (Lonza) and 0.5% Penicillin/Streptamycin (Lonza). For signaling assay GGM cells were seeded in 96 well plates (Greiner) and grown to confluence. 10.000 primary cancer cells or 2.000 knockdown PC053M cells were seeded per well on top of the GGMs in serum free medium (DMEM, Lonza) with or without the addition of 100 nM KAAD-cyclopamine (Merck Millipore). After 3 days of coculture, images were taken on a Zeiss fluorescence microscope and percentage of GFP positive cells was determined by flow cytometry on a FACSCanto II.

### RNA isolation and quantitative real-time PCR

Primary cells grown in culture were lysed in TriPure reagent (Roche) and RNA was isolated according to the manufacturer’s protocol. cDNA was synthesized using Superscript III (Invitrogen) and random primers (Invitrogen). Real-time quantitative RT-PCR was performed with SYBR green (Roche, Basel, Switzerland) on a Lightcycler LC480 II (Roche). Relative expression of genes was calculated using the comparative threshold cycle (Cp) method and values were normalized to reference gene *GAPDH*. Primer sequences used for quantitative RT-PCR amplification were as follows for GAPDH (fw 5′-GAAGGTGAAGGTCGGAGTC-3′, rv 5′- AAGGTGAAGGTCGGAGTCAAC-3′), SHH (fw 5′-CGGCTTCGACTGGGTGTACT-3′, rv 5′-GCAGCCTCCCGATTTGG-3′), IHH (fw 5′-CACCCCCAATTACAATCCAG-3′, rv 5′-CGGTCTGATGTGGTGATGTC-3′), and DHH (fw 5′-TGATGACCGAGCGTTGTAAG-3′, rv 5′-GCCAGCAACCCATACTTGTT-3′).

### *SHH* knockdown

Lentivirus was produced by transfecting HEK293T cells with the pLKO transfer construct targeting SHH (Sigma Mission library TRC clone number 0000033304) or a scrambled non-targeting control shRNA (shc002) together with the packaging plasmids pMD2.G and psPAX2 using Fugene HD (Roche). 48 h and 72 h after transfection supernatant was harvested and filtered through a 0.45 μm filter (Millipore, Germany). 60% confluent PC053M cells were transduced with the harvested virus in the presence of 5 μg/ml polybrene (Sigma) overnight. Two days after transduction cells were selected for stable transduction with 1 μg/ml puromycin (Sigma).

### Statistics

All statistical test were performed using GraphPad Prism 5 sofware, and Student’s t-test (two-sided) was used to compare grafting time between PDAC and EC patient-derived xenografts as well as the inductions measured in the Hh reporter assay.

## Results

### Tumor tissue bank establishment

To establish the appropriate tools to study pancreatic and esophagogastric malignancies, we set up a tissue collection program (biobank) in our institute. Patients with a suspicion of pancreatic or esophagogastric cancer, scheduled for operation, endoscopic procedures, or tumor biopsies from a supposedly metastatic site were asked for participation in the respective biobank. Written informed consent was obtained from all participating patients. Tissue from pancreatic cancer patients was in most cases received from resected specimen after gross examination by an experienced pathologist to confirm location and origin of the tumor. If patients were found to be inoperable due to locally advanced or metastatic disease, tissue was collected perioperatively through additional biopsies after the presence of malignancy (e.g. liver, peritoneum, distant lymph nodes) was confirmed by a pathologist.

Tissue biopsies from patients with suspicion for esophageal squamous- or adenocarcinoma were collected during diagnostic endoscopy procedure, surgery or sampling of metastatic lesions. Specimens obtained after surgical resection were processed similarly to pancreatic tissue after gross pathological examination. For biobanking purposes collected tissue material was immediately frozen in liquid nitrogen or prepared for xenotransplantation in immunocompromised mice.

In total, 63 pancreatic ductal adenocarcinoma (PDAC), 47 esophageal adenocarcinoma (EAC), and 12 esophageal squamous cell carcinoma (ESC) patients donated samples for the respective biobanks, which were started in 2011 and 2013 respectively. In the PDAC cohort from the 65 samples collected, 77% were derived from the primary tumor and 23% from metastatic sites. From two patients we were able to collect tissue from primary tumor as well as distant metastasis. The majority of the metastatic material originated from liver (13 out of 14 samples); one lymph node metastasis was collected as well. The esophagus carcinoma (EC) biobank comprises 64 tissue samples from 59 different patients, with 55% of the samples being biopsies taken from the primary tumor before neo-adjuvant chemoradiation therapy, 20% were collected from resected tumor specimen, and 25% of the tissue originated from various (strongly diverse) metastatic sites. The clinical characteristics of these two cohorts are shown in Table 1.

**Table 1:** **Clinical characteristics of Biobank patients**

	**PDAC n = 63**		**EC n = 59**
**Gender n (%)**		**Gender n (%)**	
Male	32 (51)	Male	49 (83)
Female	31 (49)	Female	10 (17)
**Age mean (±SD)**	68 (9)	**Age mean (±SD)**	64 (11)
**Stage n (%)**		**Stage n (%)**	
I	4 (6)	I	4 (6)
II	31 (49)	II	5 (8)
III	10 (16)	III	46 (62)
IV	18 (29)	IV	9 (14)
**Diagnosis n (%)**		**Diagnosis n (%)**	
**PDAC**	63 (100)	**EAC**	47 (80)
		**ESC**	12 (20)
**Tissue location n (%)**	n = 65 (63 patients)	**Tissue location n (%)**	n = 64 (59 patients)
Primary tumor	50 (77)	Biopsy tumor	35 (55)
Metastasis	15 (23)	Resected tumor	13 (20)
		Metastasis	16 (25)
**Grafting outcome n (%)**	47/65 grafted	**Grafting outcome n (%)**	61/64 grafted
Successful	12 (26)	Successful	17 (28)
Unsuccessful	26 (55)	Unsuccessful	32 (52)
Ongoing	9 (19)	Ongoing	12 (20)
**Success rate**	12/38 (32%)	**Success rate**	17/49 (35%)
(excl. ongoing grafts)		EAC	13/39 (33%)
		ESC	4/10 (25%)
		(excl. ongoing grafts)	

### Generation of xenografts from pancreatic and esophageal carcinomas

To establish patient derived xenografts from the included patients, fresh tissue pieces were immersed in Matrigel and subcutaneously implanted into the flank of immunocompromised (NSG) mice. To date 108 pieces of tissue from a total of 101 upper GI tract cancer patients have been implanted (47 PDAC, 49 EAC, and 12 ESC). From the 47 PDAC tumor pieces implanted, 12 PDAC models could be established from 11 different patients, with one patient having the primary tumor as well as the liver metastasis growing in mice. Four transplantable grafts were established for ESC, and 13 for EAC. These include two matched sets of PDXs from the same patient of which in one case biopsy material from the primary tumor as well as the metastasis was grafted, and in the other case both the pre-chemoradiation treatment biopsy as well as the post-treatment resection material growing successfully in animals. Take rates (i.e. the ability of a donor tumor to successfully be propagated in mice) were similar between the different malignancies, 32% for PDAC, 33% for EAC, and 25% for ESC (Table 1). Ongoing grafting attempts were excluded from the take rate statistics, as no clear conclusion could be drawn for these materials yet.

Grafted tumors differed quite extensively in the time required to grow to a transplantable size, ranging between 3 and 37 weeks. Esophageal carcinomas required on average 13.6 weeks (±5.8 s.d.), whereas PDAC tumors had grown to sufficient size to allow for transplantation after 20.7 weeks (±9.8 s.d.), making the esophageal tumors the slightly faster growing PDX models (p = 0.022). There was no significant difference in growth rate between the EAC and ESC xenograft tumors (p = 0.24). Clinical and pathological characteristics of all the 29 PDX models are summarized in Table 2.

**Table 2:** **Clinical and pathological characteristics of established xenograft models**

**ID**	**Gender**	**Age**	**Location**	**Stage (TNM)**	**Diagnosis**	**Diff. patient**	**Diff. xeno**	Xeno take*	**Cell lines**	**Treatment** ^**+**^
PC028	F	74	liver metastasis	pTxNxM1	PDAC	moderate	moderate	4	yes	none
PC053M	M	78	liver metastasis	pTxN1M1	PDAC	moderate	poor	12	yes	none
PC053B	M	78	biopsy tumor	pTxN1M1	PDAC	na	poor	12	yes	none
PC067	F	73	resected tumor	pT3N1M0	PDAC	poor	moderate	26	yes	none
PC072	M	65	resected tumor	pT3N1M0	PDAC	moderate	moderate	24	no growth	none
PC076	M	64	resected tumor	pT3N1M0	PDAC	poor-moderate	moderate	33	yes	none
PC084	M	83	liver metastasis	pTxN1M1	PDAC	moderate-well	moderate	11	yes	none
PC086	M	66	resected tumor	pT4N1M0	PDAC	moderate	moderate	18	na	none
PC090	F	78	resected tumor	pT4N1M0	PDAC	moderate	well	19	yes	none
PC093	M	61	resected tumor	pT4N1M0	PDAC	moderate	moderate	37	ongoing	none
PC096	F	62	liver metastasis	pTxNxM1	PDAC	moderate	moderate	29	ongoing	none
PC099	F	46	liver metastasis	cT3NxM1	PDAC	well	moderate	23	ongoing	none
EAC007B	F	50	biopsy tumor	pT3N1M0	EAC	moderate	moderate-well	10	yes	none
EAC007R	F	51	resected tumor	pT3N1M0	EAC	moderate	poor-moderate	19	yes	chemoradiation
ESC008	F	37	resected tumor	pT4N0M0	ESC	moderate	poor-moderate	3	na	chemoradiation
EAC018	M	73	resected tumor	pT3N1M0	EAC	moderate	moderate-well	16	yes	chemoradiation
EAC023	M	58	biopsy tumor	pT3N2M0	EAC	poor	poor	14	yes	none
EAC026	M	69	lymph node metastasis	pT2N2M0	EAC	poor	poor	16	yes	chemoradiation
EAC027	M	66	adrenal gland metastasis	pT3NxM1	EAC	poor	poor	14	yes	radiation
EAC031B	M	78	biopsy tumor	pT3N3Mx	EAC	moderate	poor	14	yes	none
EAC031M	M	78	liver metastasis	pT3N3Mx	EAC	moderate	moderate	17	yes	chemotherapy
EAC033	M	54	liver metastasis	pT4N2M1	EAC	poor	poor	16	yes	chemotherapy
EAC037	M	68	thoracle TH metastasis	na	EAC	poor	poor	11	yes	chemotherapy
EAC038	M	76	lung metastasis	pT3N1M0	EAC	poor	poor	28	na	chemoradiation
ESC040	F	67	pelvis metastasis	pTxN2Mx	ESC	moderate	poor-moderate	17	ongoing	chemoradiation
EAC041	M	73	biopsy tumor	pTxN1M0	EAC	moderate	moderate	3	ongoing	none
ESC043	M	58	biopsy tumor	pT3N1Mx	ESC	na	moderate-well	10	na	none
ESC049	F	66	biopsy tumor	pT3N1M0	ESC	moderate	moderate	12	na	none
EAC050	M	69	biopsy tumor	pT3N2Mx	EAC	na	poor-moderate	11	na	none

*time in weeks required for the primary patient material to grow out and be retransplanted.

^**+**^neo-adjuvant treatment before resection.

*Abbrevations*: *M* male, *F* female, *Diff.* Differentiation grade, *na* not assessed due to limited or inconclusive material, *PDAC* pancreatic ductal adenocarcinoma, *EAC* esophageal adenocarcinoma, *ESC* esophageal squamous cell carcinoma.

**Figure 1:**
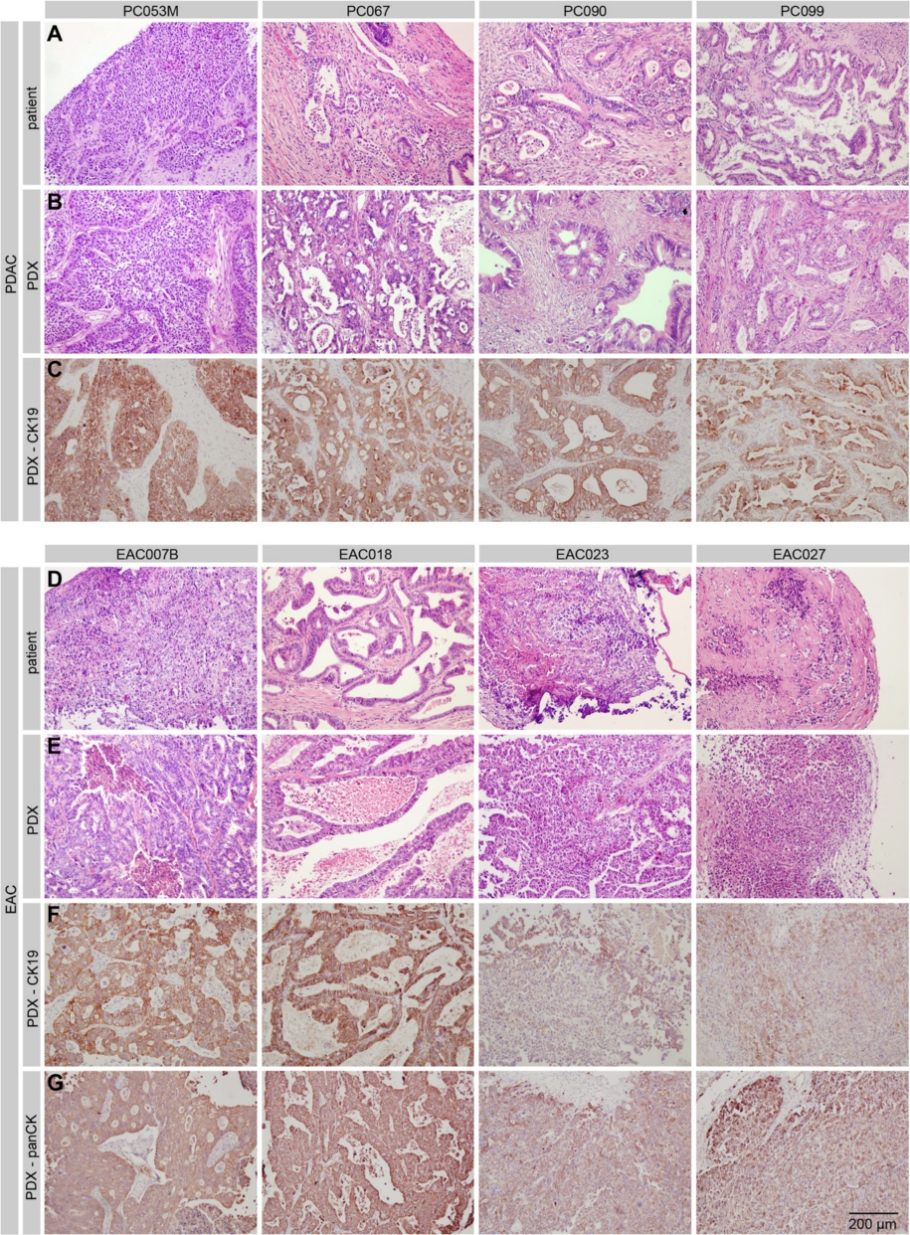
Morphology of selected primary patient tumors and the corresponding patient-derived xenograft. **A**) H&E-stained sections of original patient material and **B**) first passage xenograft tumors (p1, lower row) demonstrate overall conserved histological features. **C**) CK-19 staining using human antigen specific antibody on xenograft tumors. **D-E**) As for panels **A**-**B**, for EAC tumors and xenografts. **F**) CK-19 staining on EAC PDX tissue. **G**) Pan-cytokeratin staining on EAC PDXs. Scale bar; 200 μm.

As can be observed from the histology of 8 representative patient tumors and their derived xenografts, overall tumor morphology was well preserved in the PDXs showing strong resemblance to the respective human counterpart (Figure 1). Differentiation grades scored by a pathologist showed that tumor grade of the PDX model tended to reflect the original patient tumor (Table 2). Immunohistochemical staining specific for human cytokeratin 19 (CK19) on the PDX tissue confirmed a human origin of the epithelial cell compartment, with better staining in the moderately- to well-differentiated models compared to the poorly differentiated esophageal tumors EAC023 and EAC027 (Figure 1C,F). Similar results were obtained by using a pan-cytokeratin antibody on esophageal PDXs (Figure 1G), reflecting a downregulation of overall cytokeratin expression in poorly differentiated esophageal tumors compared to their more differentiated counterparts. Closer characterization of the stroma of a p1 PDX tumor revealed that the vast majority of the stroma was of mouse origin, indicating replacement of human stromal cells by that of the host early on in the establishment of these tumors (Additional file 1). Thus, we have been able to set up a panel of histological mimics of human upper GI tract tumors, which span the range of heterogeneity in morphology observed in patients.

### Establishment of novel primary cell lines from PDX tumors

Tumors grafted in mice allow for a strong enrichment of epithelial cells, and given the low tumor cell content often found in these tumors (most notably in PDAC), this has been instrumental in establishing cell lines. Second or higher passage PDXs at a size of around 500 mm^3^ were excised, enzymatically and mechanically dissociated, and primary lines were established. Suspensions were placed under adherent conditions and monitored for epithelial cell content by flow cytometry. Mouse cells were depleted by selective trypsin/EDTA treatment during the first passages (see Methods section).

**Figure 2:**
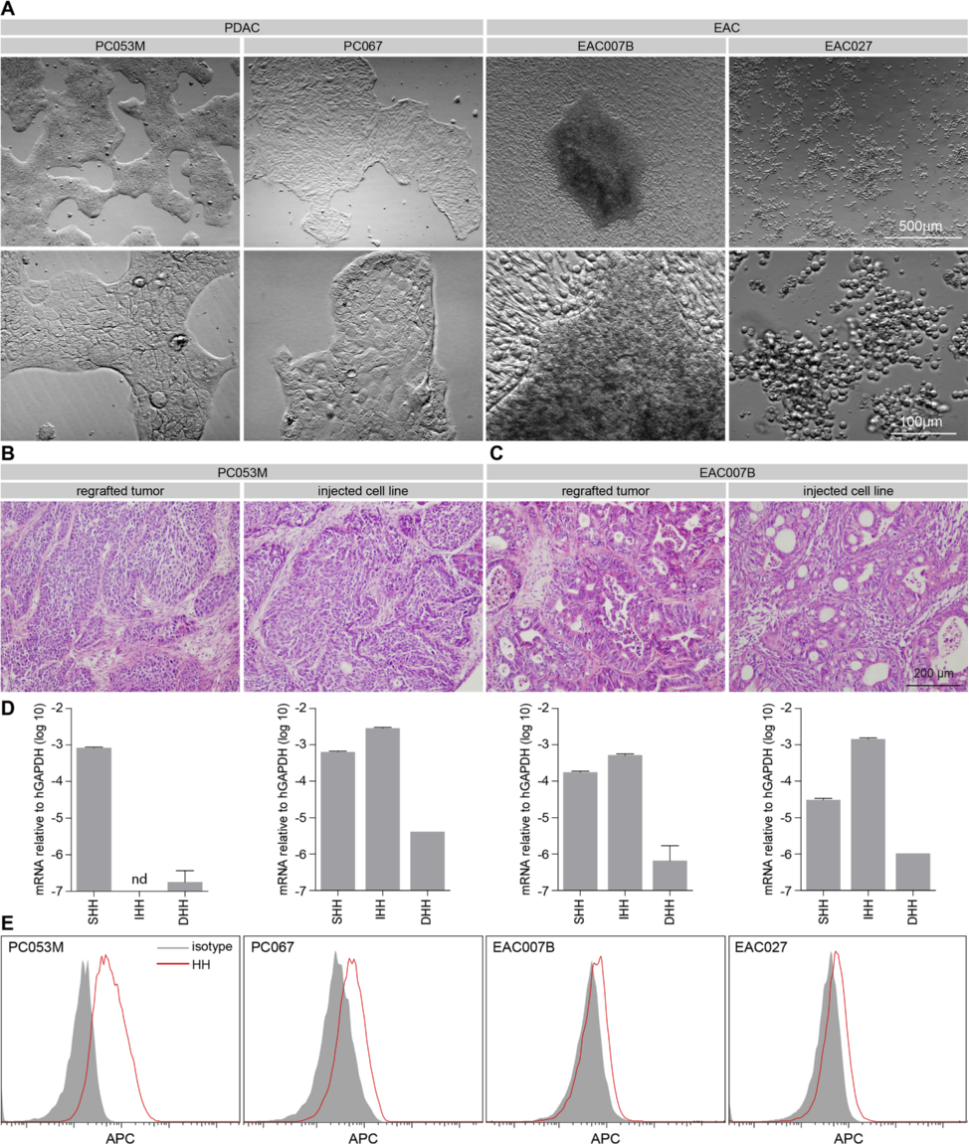
Morphology of primary cell lines and Hedgehog ligand expression **A**) Phase contrast images of cell lines generated from xenografts. **B**) H&E stained tumor grown from orthotopically injected PC053M PDAC cells (right panel) compared to p1 PDX tumor (left panel). **C**) As for panel using subcutaneous injections of EAC007B, compared to p2 PDX. **D**) Hh ligand expression determined by qPCR of respective cell lines. **E**) Surface expression of Hh protein on primary lines measured by flow cytometry using 5E1.

In case of the EAC007B cell line, it was not possible to remove the fibroblasts from the cultures, and even after more than 15 passages these cells were found to be required to support attachment and growth of the human epithelial cell clusters. Whereas most cultures (among which the PDAC cultures PC053M and PC067 shown in Figure 2A) were found to display the cobblestone morphology characteristic for epithelial cells, the esophageal line EAC027 grew semi-adherently with viable floating cell aggregates (Figure 2A), a feature previously described for established EAC lines [14]. Two of the primary lines (EAC007B and PC053M) were kept in culture for more than 12 months to test for their *in vitro* longevity. Both lines have been grown for more than 20 passages without any sign of growth decline, and rather tended to accelerate in growth after reaching approximately passage 10–15. The morphology of these cell lines was consistent in time (not shown). The histology of tumors that grew from these cells injected in mice (subcutaneously for EAC007B and orthotopically for PC053M) strongly resembled that of regrafted PDX tumors (Figure 2B-C).

### Hedgehog ligand expression in upper GI tract tumors

Hedgehog ligands and activation of its downstream pathway have been implicated in the genesis and progression of several diseases of the gastrointestinal tract [2]. To formally assess the expression of these ligands in our primary cultures, transcript levels were measured by qRT-PCR and robust expression was found for the *Sonic* (*SHH*) and *Indian Hedgehog* (*IHH*) paralogs (Figure 2D). Protein levels on the surface of these cells were confirmed by flow cytometry using the 5E1 antibody (Figure 2E). The limited expression found for *Desert Hedgehog* (*DHH*) fits with its very restricted expression pattern in healthy organisms [15], and the expression of the *IHH* homolog in EAC cells is in line with profiling of ligands in patient tissue [16]. Expression of both *IHH* and *SHH* paralogs has been reported in PDAC biopsies and cell lines [2], but most studies have typically focused on the better characterized SHH protein. The presence of one or more Hedgehog ligand implies that there is selective pressure on tumor cells to express Hedgehog, but that the exact protein expressed is not critical and that either *IHH*, *SHH,* or both will suffice. It also suggests that in the divergent evolution of the two paralogs some of their regulatory mechanisms are coevolved, and that these mechanisms exist throughout the organs of the upper GI tract.

**Figure 3:**
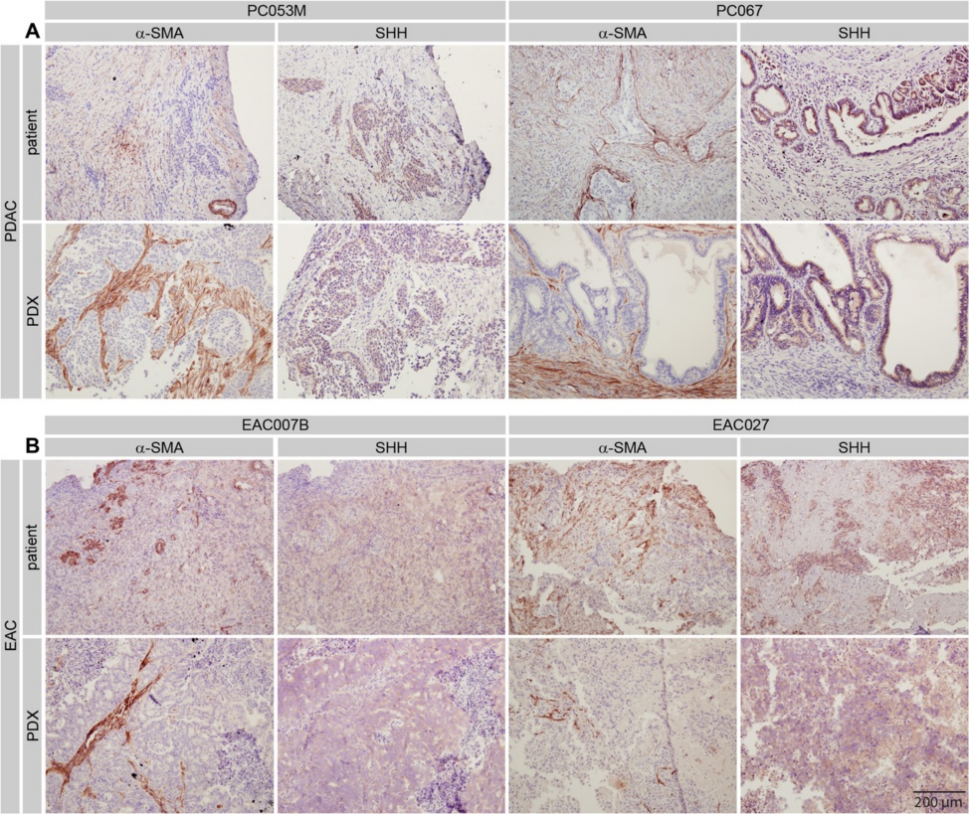
Hedgehog ligand expression and stromal activation in xenograft tumors **A**) Immunohistochemical staining shows expression of Hedgehog ligand in the PDAC patient material as well as in matching xenografts. Surrounding tissue is positive for stromal activation marker alpha smooth muscle actin (α-SMA). **B**) As for panel A, on the esophageal cancer primary tumors and xenografts. Shown are first passage grafts (p1).

In agreement with the expression data from the primary cell lines, immunohistochemistry for Hedgehog ligands (presumably SHH) confirmed a widespread expression in the epithelial compartment of the human tumors and this was conserved in the PDXs (Figure 3). Generally stronger staining was observed in PDAC (Figure 3A) than EAC tissue (Figure 3B).

Stromal activation marked by alpha smooth muscle actin (α-SMA) was apparent in all tumors tested, but we did observe that the non-epithelial areas were larger in the patient tumors than they were in the PDXs, specifically for PDAC. This possibly contributed to the higher staining intensity for α-SMA observed in the PDXs derived from these patients, as a consequence of a higher tumor/stroma cell ratio in these tumors. Differences were also observed between EAC and PDAC PDXs, the latter showing typically larger areas of α-SMA positivity. This reflected the known high stromal content of PDAC tumors in general [17], and as found in our patient tumors. These differences in stromal activation, as well as SHH staining intensity, were consistently found over several consecutive passages (Additional file 2).

### Paracrine Hedgehog signaling in cell lines

As mentioned above, pancreatic tumor cells express increasing amounts of SHH as they progress towards fully established PDAC, however, they are unresponsive to the ligand themselves. Instead, the ligand signals to responsive cells in the adjacent stroma, activating the pathway in the non-malignant compartment [18,19]. A similar signaling model has been shown in Barret’s esophagus, a pre-malignant condition that may progress towards adenocarcinoma, but there are no conclusive studies addressing the mode of Hedgehog signaling in EAC yet [16]. Hh pathway inhibitors are already successfully used to treat patients with basal cell carcinomas (BCC), and are currently being tested in clinical trials for the use in several other malignancies [20]. Although these efforts indicate a certain level of (perceived) importance of this pathway in human cancer, unfortunately preliminary reports have been disappointing. Especially in patients with advanced pancreatic cancer, which is probably due to our lack of understanding the stromal response.

We have recently developed a fibroblast reporter cell line in order to quantitatively assess the paracrine signaling potency and range of cancer cell-derived Hedgehog ligands. These cells (GBS-GFP MEFs; denoted GGM) express eGFP under the control of eight concatemerized Gli-binding site motifs, which are recognized by the GLI transcription factors mediating the downstream transcriptional response following canonical Hedgehog pathway activation. These reporter cells respond to known Hh pathway stimulating agents as well as overexpressed and tumor cell-derived Hedgehog proteins. This activation can be blocked by administering either Hh blocking antibodies or small molecule inhibitors [21].

**Figure 4:**
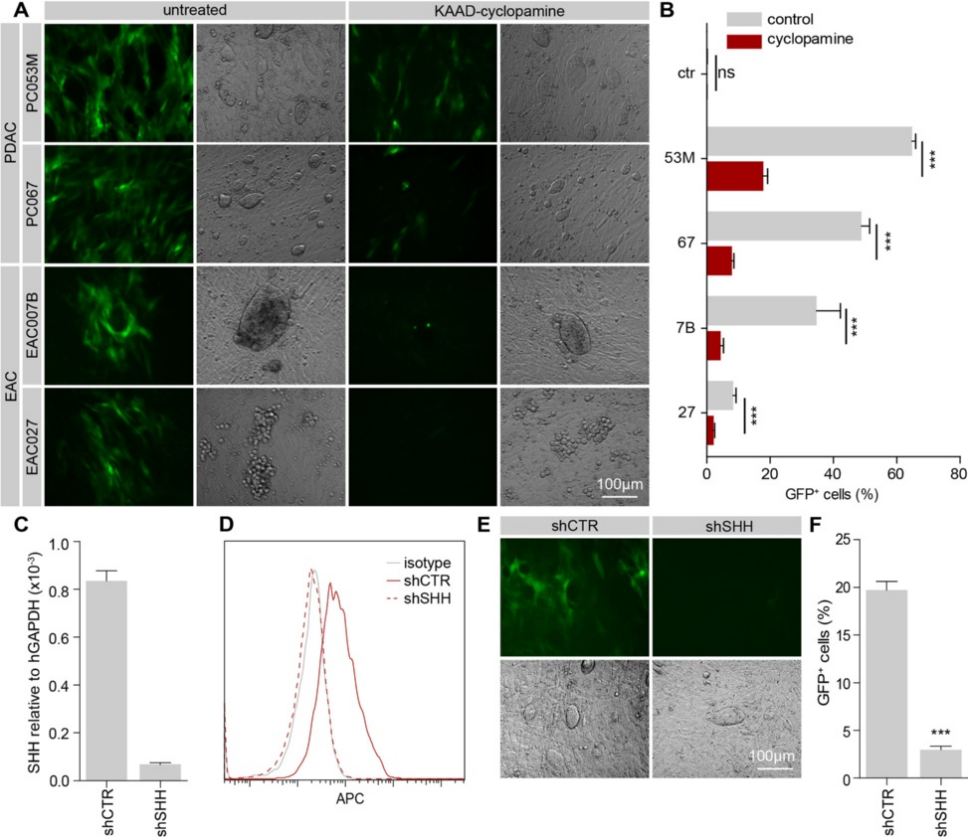
Hh expression is maintained in primary cell lines and can activate reporter cells in a gradient **A**) The fibroblast Hh pathway reporter cell line GGM was cocultured with indicated primary cell lines for 3 days. Representative fluorescence and brightfield images show GFP expression in reporter cells adjacent to cancer cells. Administration of Hh inhibitor KAAD-cyclopamine (100 nM) strongly reduced pathway activation. **B**) Quantification of GFP positive GGMs following coculture by flow cytometry. ns, not significant; *** p < 0.001. Percentage of GFP positive GGMs in the absence of tumor cells was 0.135 ± 0.05%, and 0.141 ± 0.06% in the presence of KAAD-cyclopamine. **C**) qPCR of *SHH* transcript in shCTR and shSHH PC053M cells relative to *GAPDH*. **D**) PC053M knockdown cells stained with anti-HH antibody 5E1(red) or isotype control (grey). Representative histogram overlay of flow cytometry experiment is shown. **E**) GGM coculture with knockdown cells imaged after 3 days. **F**) Quantification of GFP positive GGMs after coculture by flow cytometry shows strong reduction of paracrine pathway activation after knockdown of *SHH* in PC053M cells. *** p < 0.001.

Primary cancer cells were seeded on top of a monolayer of GGMs and cocultured for a period of three days in the absence of serum to allow for full pathway activation and accumulation of eGFP in the reporter cells (Figure 4A). Strong activation of eGFP expression could be observed in reporter cells in close proximity to the tumor cells, but not in those cells at larger distances from the ligand source, a function of the hydrophobic properties of Hedgehog protein, which is apparently consistent between different tumor types. Reporter activation could be ablated by treating cocultures with the Smoothened antagonist KAAD-cyclopamine. Flow cytometry was used to quantify the percentage of GFP-positive cells in the cocultures. All tested primary cell lines were able to activate GFP expression in the reporter cells to different magnitudes, and this activation was sensitive to cyclopamine (Figure 4B). Generally, PDAC cells were more potent in activating Hh response than the EAC lines, which matches the expression levels of the *HH* paralogs determined in Figure 2.

For a more targeted approach and to test if our primary cell lines are amenable to genetic manipulation, we decided to perform stable knockdown of *SHH* in the PDAC line PC053M, as these cells do not express the other *HH* paralogs that could confound interpretation of the results. PC053M cells were lentivirally transduced with a control or *SHH* targeting shRNA, and after selection with puromycin, target gene transcript levels were measured by qPCR (Figure 4C). Successful reduction of protein levels was assessed by Hh surface staining using flow cytometry (Figure 4D). Coculture of these cells with GGMs showed a strongly diminished capacity of *SHH* knockdown cells to active Hh response (Figure 4E,F). These results demonstrate that the primary cells are amenable to genetic manipulation and provide a powerful tool to study disease biology.

## Discussion

Despite encouraging progress in the treatment and prognosis of most tumor types, esophagogastric and pancreatic cancer are almost as lethal as they were decades ago [22]. There is a clear drive to find new drugs to combat these tumors, but the numerous candidate agents that showed promising therapeutic efficacies in preclinical studies have failed to improve outcome in clinical trials [20]. A number of factors can be held accountable for these failed trials, including the obvious problem that several preclinical models have not managed to recapitulate the heterogeneity observed in the relevant patient population [23,24]. In addition, they need to be endowed with the intrinsic and acquired resistance mechanisms that are a result of tumor evolution over the course of many years of disease progression in the patient.

To match the relevant tumor biology, we have generated a panel of PDXs that mirror the (immuno)histochemical and biochemical features of human disease. For three patients, this panel contains matched xenografts from the primary tumor and metastasis or post-chemoradiation material. These will provide excellent tools to study tumor development and evolution over time, as well as to investigate changes in the primary tumor in response to therapy. Furthermore, we created matching cell lines amenable to genetic manipulation like lentiviral transductions and the knockdown of genes. Upon injection, these cells formed tumors histologically similar to those grown from grafted tumor pieces. Having such cultures available greatly broadens the possibilities for studies that genetically address the functional relevance of potential targets in tumors that closely resemble the patient tumor. In this study we demonstrate the silencing of one morphogen as an example, however, the availability of a genome-wide shRNA library enables us to target any gene, pending viability of cells knocked down for it.

PDX models are not without limitations. For instance, we have observed that quite long periods of time are required for grafted tumors to grow, especially for PDACs. This limits the use of these models as avatars to guide (neo)adjuvant treatment or first line treatment for metastatic disease. We were able to generate cell lines from 60% of xenografts, but have not been able to correlate this to clinical parameters like tumor stage, grade or patient survival. PDXs that were passaged subcutaneously did not yield the metastases that are characteristics of the diseases they are derived from, and for this orthotopic transplantation is necessary [24,25]. Despite these caveats, having an extensive range of PDXs to perform experimental work on is preferable over having to rely on a limited number of genetically defined mouse models for preclinical experiments.

Our biochemical data reveal a remarkable congruence in the expression of Hedgehog ligands between the different tumors, PDXs and cell lines. Berman *et al.* had previously demonstrated that Hedgehog ligands were expressed in tumors throughout the entire gastrointestinal tract, and that this was functional [2]. Although later studies did show that some of the conclusions in the paper were possibly overstated, our results do indicate that the expression and distribution of Hedgehog ligands are conserved in the upper GI tract tumors tested.

Despite the shared expression of Hedgehog ligands between EAC and PDAC, some differences in the levels do exist and these could be caused by the dissimilarities between Hedgehog signaling in the healthy pancreas and esophagus. In the pancreas, SHH is absent during development as well as adult life, but expression is activated after malignant transformation through mechanisms that are not entirely understood. In the esophagus, SHH expression during development is needed for proper foregut separation and the formation of squamous epithelium [26]. Moreover, in adult tissue Hedgehog ligand expression stimulates proliferation of epithelial precursor cells [10]. The exact role of Hedgehog ligands during malignant progression in this organ, or how expression is regulated here, remains to be determined. The latter is further complicated by the apparent contradictory signaling mode for Hedgehog in adenocarcinomas versus squamous cell carcinomas. Hedgehog expressing adenocarcinomas were described to signal to the adjacent stroma [16] as well as in an autocrine fashion [9], however, also ligand independent pathway activity was observed [27,28]. Squamous cell carcinomas on the other hand appear to arise and be maintained by autocrine pathway activation in the epithelium [8,10]. Our primary cell line models at the moment are restricted to the adenocarcinoma type, but it will be interesting to include and test squamous carcinoma cells for their paracrine signaling activity.

In light of the paracrine signaling by Hedgehog ligands, the sustained expression of these proteins in culture is remarkable. For PDAC it is well know that the responsiveness resides in cells from the stroma, which are of course absent from the epithelial monocultures. In addition, the expression of these proteins are not known to rely on gene duplications, promoter mutations or other genomic alterations and therefore, a plastic regulation at the level of epigenetics or more upstream could be driving this expression. The retained production of this ligand in culture does suggest it to be somehow hard-wired in these cells.

## Conclusions

Therapeutic strategies aiming at targeting and ablating the reactive tumor stroma show promising results in a group of pancreatic cancer patients [29], however the molecular mechanisms and exact tumor-promoting and -inhibiting functions of the stroma are still not very well understood. The established PDXs that retain a reactive stromal microenvironment, and the availability of matching primary cell lines, will enable future studies to address their responses to different treatment modalities. This will not only aid in the development of new treatment strategies, but hopefully also in gaining mechanistic insight in how some tumors respond to certain regimens and others do not. This, in turn, can shed light on the biology underlying these malignancies.

## Additional files

Additional file 1:
**Human stroma is replaced by cells of murine origin early in PDX outgrowth.** Flow cytometric analysis of patient-derived xenograft PC084 passage 1. Tissue was enzymatically dissociated and single cell suspension was stained with FITC labelled anti-human EpCAM antibody or biotin labelled anti-mouse H2Db antibody (BD Bioscience, 1:100) followed by incubation with streptavidin-APC (Invitrogen, 1:500). Single and double stainings reveal around 70% human epithelial cells and 30% cells of murine origin. Stainings indicated in panels. Additional file 2:
**Hedgehog ligand expression and stromal activation in xenograft tumors over several passages.** A) Immunohistochemistry for Hedgehog ligand (SHH), and the stromal activation marker alpha smooth muscle actin (α-SMA) in the PDAC xenografts over passages p2-4. B) As for panel A, on the EAC xenograft passages p2-4. Earlier passage and original patient tumor are shown in Figure 3. na, not assessed.

## Abbrevations

HH: Hedgehog
PDAC: Pancreatic ductal adenocarcinoma
ESC: Esophageal squamous cell carcinoma
EAC: Esophageal adenocarcinoma
EC: Esophageal cancer
GGM: Gli-binding site-GFP-mouse embryonic fibroblasts
GI: Gastrointestinal
PDX: Patient derived xenograft
GAPDH: Glyceraldehyde 3-phophate dehydrogenase
