# Supplementary figures and images for: Establishment of patient-derived xenograft models and cell lines for malignancies of the upper gastrointestinal tract
Source: J Transl Med. 2015 Apr 11;13:115. doi: 10.1186/s12967-015-0469-1 (PMC4419410; doi:10.1186/s12967-015-0469-1)

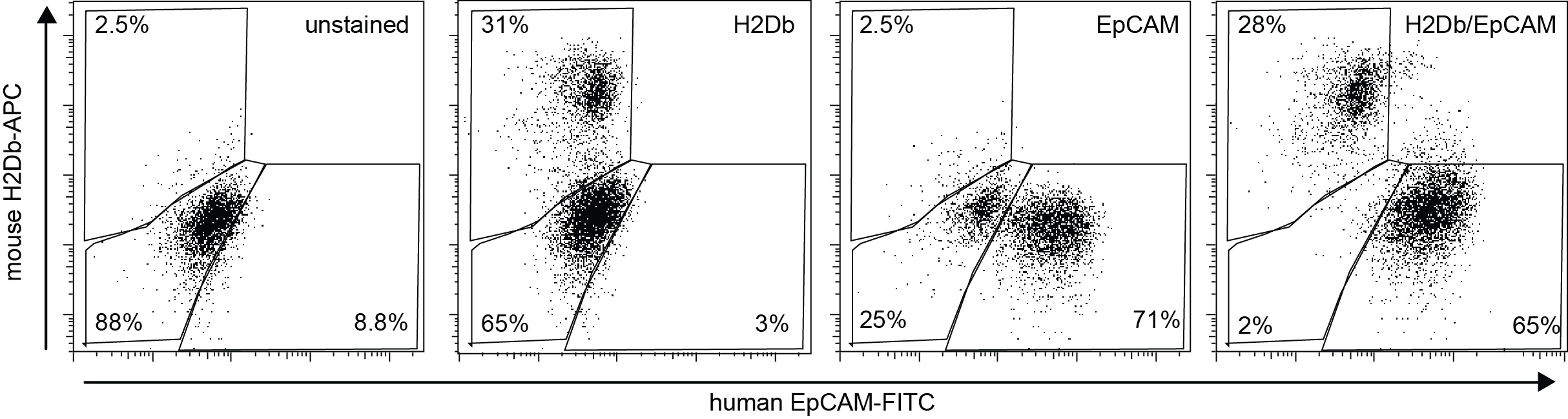

Supplement: Additional file 1: — Human stroma is replaced by cells of murine origin early in PDX outgrowth. Flow cytometric analysis of patient-derived xenograft PC084 passage 1. Tissue was enzymatically dissociated and single cell suspension was stained with FITC labelled anti-human EpCAM antibody or biotin labelled anti-mouse H2Db antibody (BD Bioscience, 1:100) followed by incubation with streptavidin-APC (Invitrogen, 1:500). Single and double stainings reveal around 70% human epithelial cells and 30% cells of murine origin. Stainings indicated in panels. [file 12967_2015_469_MOESM1_ESM.png]

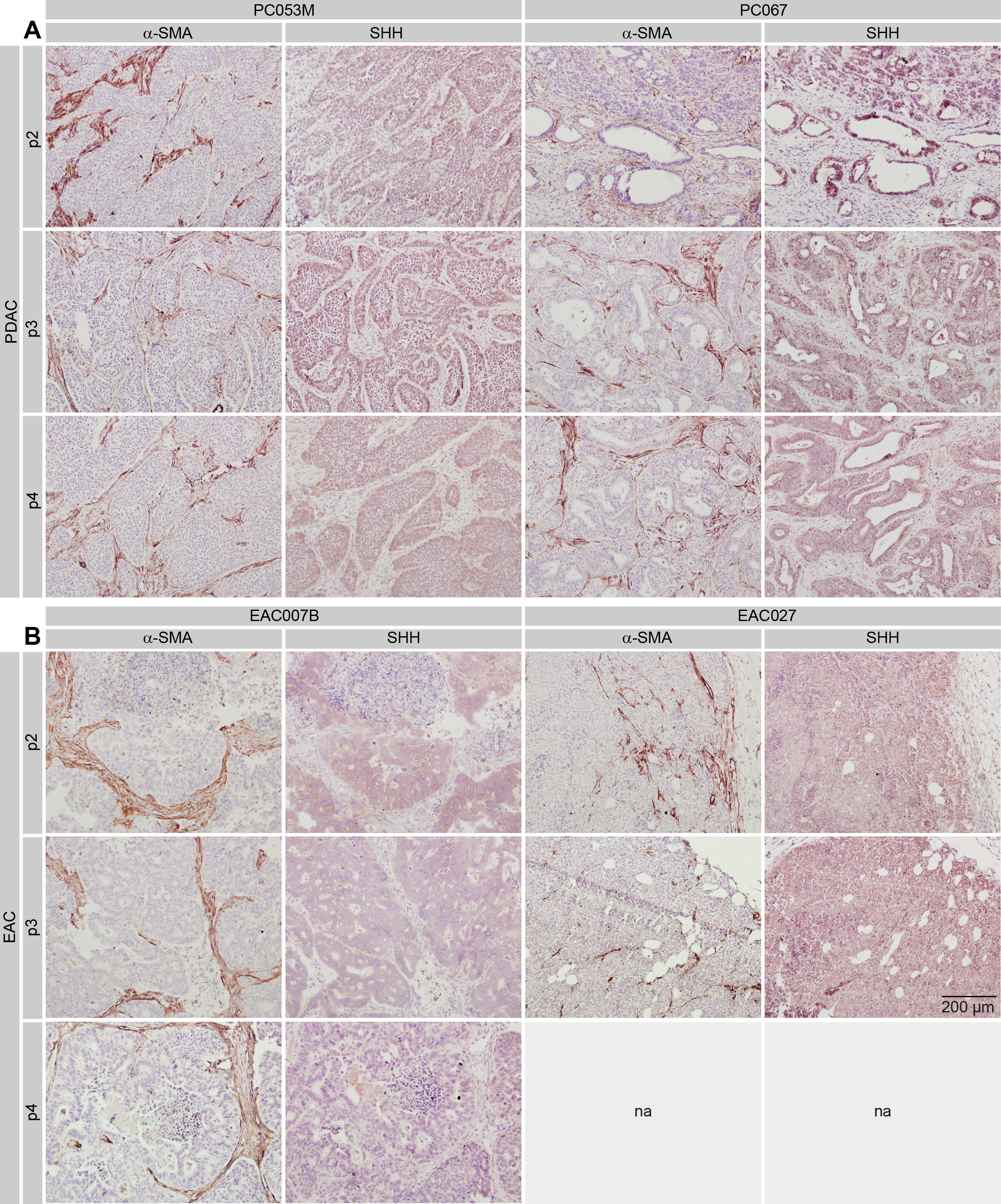

Supplement: Additional file 2: — Hedgehog ligand expression and stromal activation in xenograft tumors over several passages. A) Immunohistochemistry for Hedgehog ligand (SHH), and the stromal activation marker alpha smooth muscle actin (α-SMA) in the PDAC xenografts over passages p2-4. B) As for panel A, on the EAC xenograft passages p2-4. Earlier passage and original patient tumor are shown in Figure 3. na, not assessed. [file 12967_2015_469_MOESM2_ESM.jpeg]
